# A Small-Scale Hopper Design Using a Power Spring-Based Linear Actuator

**DOI:** 10.3390/biomimetics8040339

**Published:** 2023-08-01

**Authors:** Seon-Gyo Yang, Dong-Jun Lee, Chan Kim, Gwang-Pil Jung

**Affiliations:** 1 Department of Mechanical and Automotive Engineering, SeoulTech, Seoul 01811, Republic of Korea; usultgiu@gmail.com (S.-G.Y.);; 2 School of Mechanical and Aerospace Engineering, Seoul National University, Seoul 08826, Republic of Korea

**Keywords:** bio-inspired robot, hopping robot, linear hopping

## Abstract

Hopping locomotion has the potential to enable small-scale robots to maneuver lands quickly while overcoming obstacles bigger than themselves. To make this possible, in this paper, we propose a novel design of a high-power linear actuator for a small-scale hopper. The key design principle of the linear actuator is to use a power spring and an active clutch. The power spring provides a near constant torque along the wide range of output displacement. The active clutch controls the moving direction and operation timing of the linear actuator, which enables the hopper to take off at the right timing. As a result, the hopper has a size of 143 mm, a mass of 45.9 g, and hops up to 0.58 m.

## 1. Introduction

Mobile robots can survey the environment and perform detection missions on behalf of humans. Among mobile robots, small and lightweight robots are less expensive to build and more energy efficient. Their size and weight also make them easy to carry, allowing them to be paired with humans or other carrier robots. These features make them versatile for many specialized purposes and applications. However, due to their small size, small mobile robots are highly affected by terrain and obstacles and need a way to overcome them. Jumping locomotion overcomes the limitations imposed by their small size and is an effective locomotion method for travelling over discontinuous paths and obstacles. Several studies have attempted to increase jumping performance by amplifying the power output of the motor and have used elastomers to do so [1,2,3,4,5,6,7].

Mechanisms for storing and releasing energy in elastic materials are an effective way to improve jumping performance without excessively increasing weight and size. Jumping robots developed to date have demonstrated successful performance. However, the process of storing energy for power amplification takes tens of seconds or longer, making agile movement difficult. To solve this problem, recent studies have been conducted to achieve hopping. Hopping locomotion involves a series of jumps, with some of the impact of the landing being reabsorbed and used for the next hopping. The kangaroo rat is a good example of hopping on the small-scale. Among kangaroo rats, Dipodomys deserti can travel a distance of 0.28 m (2 BL) horizontally and 0.15 m (1 BL) vertically in a single hop. When confronted with a predator, they can jump vertically up to 0.4 m with high acceleration [8,9,10]. Furthermore, kangaroo rats reabsorb about 14% of their energy on each landing and use it for the next hop [11].

To achieve kangaroo rat-like hopping locomotion, a high-powered drive design is essential. To achieve this, several hopping robots have been developed recently. Salto adopts a power modulation approach to maximize its hopping performance. An optimized eight-bar linkage was designed for power modulation. During the first half of the jump, the energy from the motor is stored in the rubber tube. In the second half, the energy stored in the rubber tube and the torque of the motor are simultaneously released in an explosive burst, resulting in a high-performance continuous jump. The trajectory of the leg tips is also designed to be close to a straight line, while still passing through the center of mass, so that there is very little rotation of the entire robot. Additionally, Salto uses a mechanical advantage through an 8-bar linkage and achieves high vertical agility through series elastic actuation [12,13,14].

Dipo is a small hopping robot with a four-bar linkage leg structure and an active clutch. Dipo has a total height of 5 cm and weighs about 45 g in the stance phase. To make it small and lightweight, the robot used a power spring that requires less torque to store energy compared to a typical spring. In addition, the power spring requires less space to store energy compared to a large total displacement, allowing for a compact design. Dipo is capable of hopping up to 54.9 cm using the active clutch and power spring, which is 11 times the length of the robot in stance [15]. The inverted cam-based hopper is a robot that has legs with a six-bar linkage and uses an inverted cam to modulate power as a mechanism to store and release energy. The inverted cam consists of two cranks and a cam-shaped shell. In addition, the crank is composed of two links and a torsion spring, and when storing energy, the energy is stored in the torsion spring inside the crank in the form of a parallel elastic actuator (PEA) by the cam-shaped shell. The crank then leaps out of the shell, releasing energy like a series elastic actuator [16].

Nasonov et al. shows a hopping robot using computational design of closed-chain linkages [17]. Through morphological computation, the robot is designed to have minimum control effort needed to excite and stabilize. As a result, the robot uses two low-performance actuators and achieves hopping and running. Chen et al. proposes an origami-inspired jumping leg that can directly tune the leg’s stiffness [18]. Stiffness of the jumping leg is tuned through a laminated design and fabrication method, providing the option to adjust both the stiffness coefficient and the nonlinearity of the force-displacement curve. As a result, the properly tuned jumping leg showed an increase of 19% improvement in peak power output.

There are also hopping robots that use a linear structure as shown in Table 1. Hopping robots based on rotational links have asymmetrical leg structures with respect to the coronal plane. When a linkage-based hopping robot takes off, each link rotates around its joint, resulting in a nonlinear trajectory for the foot, which increases the rotational momentum of the body. In order for the robot to take off stably, the foot must follow a straight trajectory and pass through the center of gravity of the robot to minimize the rotational momentum of the body. Since these robots are structurally designed to take off with a straight trajectory, it is relatively advantageous to reduce the rotational momentum.

REBO hopper is a hopping robot that uses the REBO pattern in the origami structure as a spring to store and release energy. The hopper consists of three REBO springs connected in parallel to form one compliant leg, and each spring is controlled by an individual motor, pulley, and tendon, giving it three degrees of freedom (DoF). The compliant leg changes the position of the foot as the length of each tendon changes, and this allows the hopper to perform fore-aft hopping as well as vertical hopping [19].

Another hopping robot uses power modulation based on auxetic tubular springs fabricated using thermoplastic polyurethane (TPU) material and a 3D printer. The robot consists of three springs, a motor, a tendon, and a foot. The motor controls the length of the tendon, and the tendon stores or releases energy to the spring. An auxetic spring has a negative Poisson’s ratio, which means it gets stiffer as it stores energy, and this feature makes it more efficient than a linear spring. By adjusting each tendon, the robot can change direction and has a total of one degree of freedom in the vertical direction and two DOFs in the forward direction [20].

Disney LEAP is a hopping robot composed of voice coil actuators and parallel springs. The voice coil actuator has low inertia due to its lightweight coil and has no gearing parts, resulting in virtually no friction. The spring connected in parallel with the actuator reduces the force and power required for hopping and allows the voice coil to store energy directly in the linear spring. To maximize the robot’s hopping performance, a strategy is employed to not pre-compress the spring in mid-air during hopping, but to apply voltage only when the spring is compressing and extending [21].

**Figure 1:**
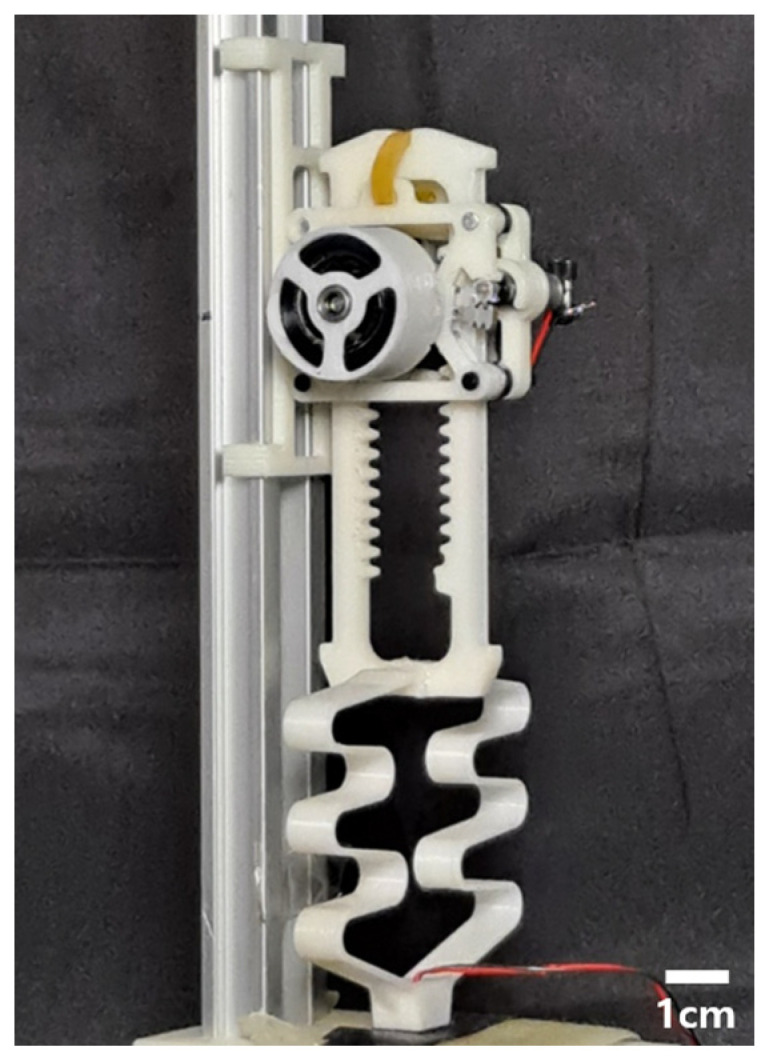
The proposed hopping robot. The robot has a height of 143 mm and weight of 46 g.

The mentioned robots above have shown successful hopping locomotion in terms of hopping height shown in Table 1; however, there still exists room to improve performance. In this paper, we propose a small-scale and lightweight linearly hopping robot, as shown in Figure 1. To improve the robot’s hopping performance, a powerful linear actuator is newly developed. The key components are a power spring, specially designed rack-and-pinion, and an active clutch. The power spring is suitable for dividing the desired amount of energy into as many parts as needed because the torque required to charge the elastic energy is small, and it has a central flatter section where the magnitude of the output torque is almost constant. The specially designed rack-and-pinion structure is created to make a linear motion. Linear actuation is advantageous to minimize the rotational momentum during hopping. If the reaction force always follows a straight line and is designed so that the reaction force passes through the mass center, the momentum can be reduced to almost zero during hopping. An active clutch enables the pinion gear to contact the left or right side of the rack, controlling the direction of motion of the rack. With these key components, the proposed hopper has a size and weight of 143 mm and 45.88 g, respectively, and a maximum hopping height of 58 cm.

**Table 1:** Small-scale hopping robots using linear actuators.

Robot	Size (cm)	Mass (kg)	Actuator	Energy for a Stroke (J)	Stroke Power (w)	Hopping Height (Body Length (cm))
LEAP [21]	32 (estimated)	1.46	Voice coil actuator	-	-	0.27 (8.5) (estimated)
REBO hopper [19]	23	2.53	Tendon-driven REBO pattern-based springs	1.23	20	0.24 (5.6)
Auxetic hopper [20]	30 (estimated)	2.5	Tendon-driven auxetic tubular springs	1.08	18	0.2 (6) (estimated)
Proposed robot	14.3	0.046	Power spring and active clutch	0.38	19.1	3.66 (52.3)

This paper is presented in the following order. The design section describes the structure of the hopping robot, its core operating principles, and the hopping timing control. In the modelling section, kinematic modelling is performed to analyze the hopping motion, which ends in a very short time. Finally, the experimental section demonstrates the hopping performance through performance tests of the built hopping robot.

## 2. Design

The main objective of the proposed hopping robot is to ensure that the trajectory of its feet is straight in order to minimize the rotation of the robot body. At the same time, designing the reaction forces to pass through the robot’s center of gravity is important. We first focus on the straight trajectory of the hopping robot’s feet, and linear actuators can easily achieve this. However, we need a linear actuator that is lightweight, compact, and high powered. We design a linear actuator with a rack-and-pinion structure using a power spring-based series elastic actuator to produce the output required for hopping with a small motor.

Since the spring can only rotate in one direction, we use an active clutch and a specially designed rectangular rack structure for bi-directional drive output. The drive direction of the rectangular rack changes depending on the side that the pinion touches. The active clutch allows the pinion to selectively change the side that the rack touches. As a result, the design allows for bi-directional drive through the active clutch mechanism.

### 2.1. Hopping Mechanism

**Figure 2:**
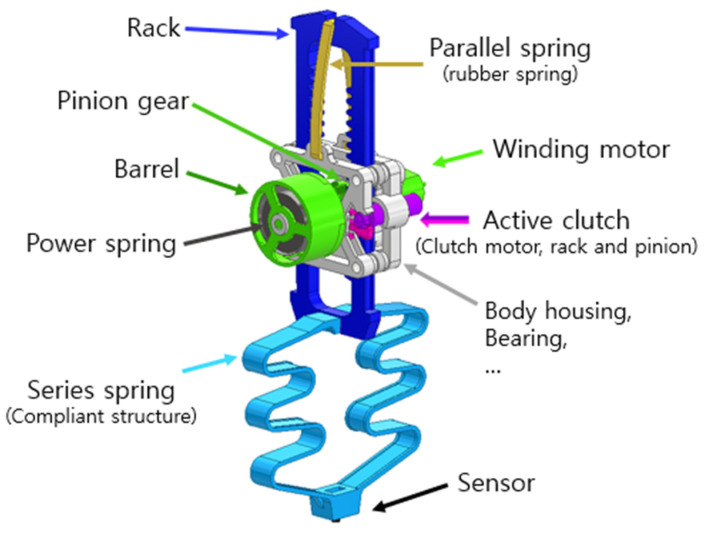
Overall design of the proposed hopping robot.

Figure 2 shows an overall view of the hopping robot. It is divided into two parts: the actuator and the hopping legs. The hopping leg is basically in the form of a rack. The rack receives power from the actuation part and performs reciprocating motion up and down. At the lower end of the rack, there is a compliant structure connected in series, which acts as a passive spring. The passive spring installed at the feet absorbs the impact of landing in the form of elastic energy, which is used for the next hopping. There is another passive spring connected in parallel at the top of the rack. The top passive spring also absorbs the energy of the landing and uses it for the next hopping. At the end of the leg, there is a sensor to detect contact with the ground. Table 2 shows the components and mass budget used in the hopper.

The actuation part is located in the center of the robot and consists of a power spring, barrel, pinion gear, and winding motor, as shown in Figure 2. The actuator is based on the power spring, which transmits elastic energy to the rack. The power spring is located inside the barrel and uses the winding motor to store elastic energy. The energy stored in the power spring is stored or released by the active clutch. Depending on the direction of motion of the active clutch, the actuator can move the rack up or down.

**Figure 3:**
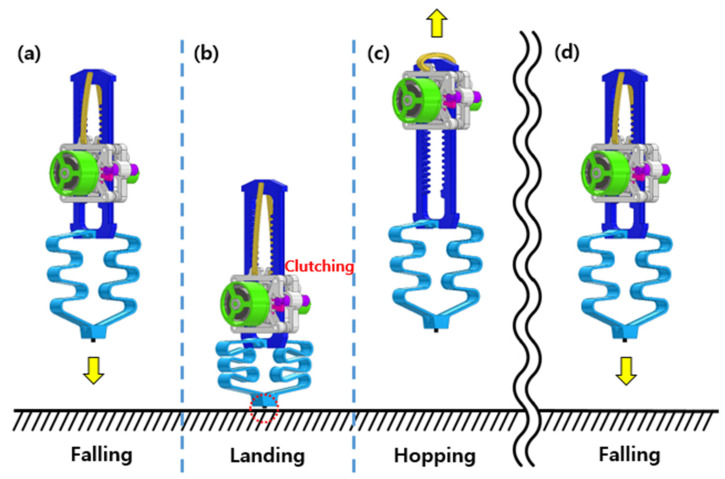
Overall hopping process. (**a**) Falling, (**b**) landing, (**c**) take-off, and (**d**) falling.

Figure 3 shows the overall hopping process. In Figure 3a, the hopper lands from the air to the ground. In Figure 3b, the landing occurs, and energy is stored in two passive springs. The passive spring connected in parallel stores energy as it stretches, and the passive spring connected in series stores elastic energy as it compresses. During the landing phase, a sensor at the end of the foot detects ground contact, which triggers the active clutch. The active clutch moves the actuation part containing the power spring, allowing the energy to be released. Finally, the rack moves downward, and the robot performs a leap as shown in Figure 3c. This process occurs repeatedly to perform hopping.

### 2.2. Power-Amplifying Actuator

Jumping robots have different amounts of time to store energy depending on the purpose of the jump. Unlike the robots that are intended to move in a single jump, the robots that are intended to move by hopping continuously have a limited amount of time to store energy. Hopping robots require high-powered actuators because they need to re-leap within a short contact time with the ground. In this paper, a power spring is utilized as a key element to achieve continuous high-power drive.

**Figure 4:**
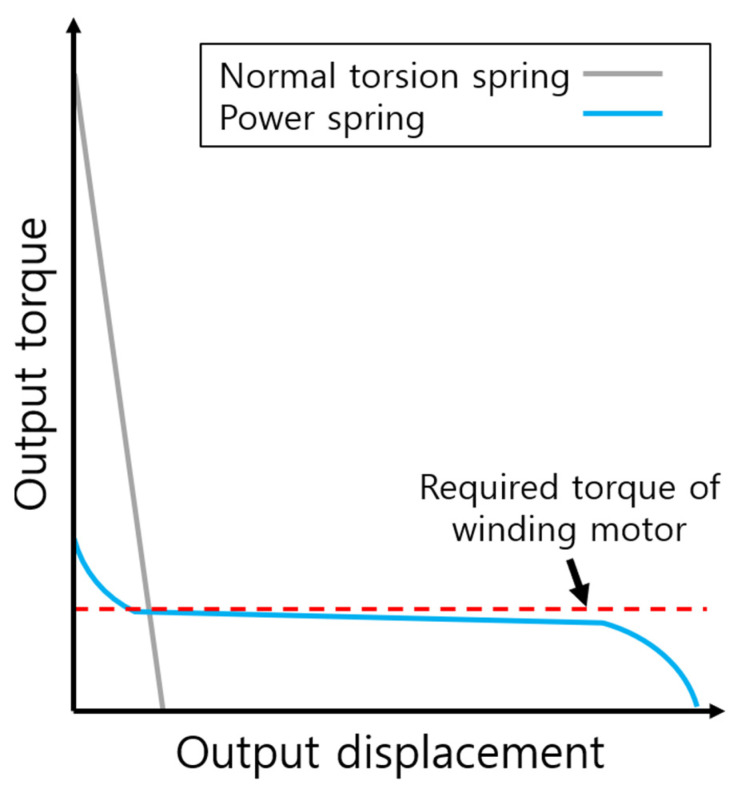
Output torque curves of a normal torsion spring and a power spring. Power spring has the central, flatter section showing constant torque.

Figure 4 shows the torque curve as a function of displacement for a typical torsional spring and a power spring. A typical torsional spring has a maximum torque at the beginning, and the torque decreases linearly with displacement. In order to store energy in a torsional spring, a motor capable of producing a higher torque than the maximum torque is required, which requires a high reduction ratio in the motor or a strong torque in the motor itself. However, increasing the reduction ratio of the motor reduces the actuation bandwidth, which is unfavorable for hopping, and using a motor with high torque increases the weight and size, which negatively affects the jump.

We use a power spring to amplify the output while using a small and low-speed motor. As shown in Figure 4, the power spring has a very wide range of angular displacement [22]. In addition, the increase rate of torque to displacement is lower than that of a typical spring, and the torque output is almost constant, especially in the central flatter section. In other words, as long as the winding motor is higher than the torque in the central flatter section, there is no problem storing energy in the power spring. Furthermore, because the power spring stores energy in a wide range of displacements, it is suitable for using as much energy as desired by splitting it into sections. In terms of volume, it also allows for a compact design because it stores energy within a limited space.

**Figure 5:**
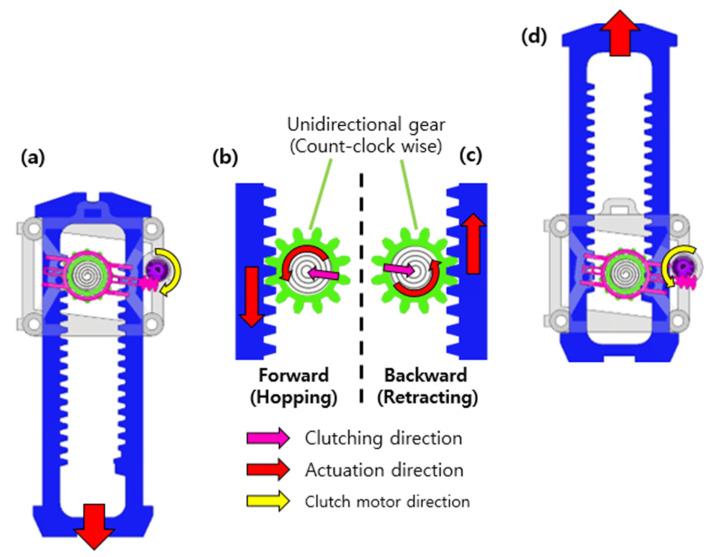
Working process of the active clutch. (**b**) The clutch contacts the left side of the rack (**a**) and the rack moves downward. (**c**) The clutch contacts the right side of the rack and (**d**) the rack moves downward.

The radius of the unidirectional gear in Figure 5 has been selected as 6 mm. For a single hop, the power spring releases the stored energy during 425.9 degrees. Therefore, at least 44.6 mm travel length is required to the rack. Finally, the rack is designed to have 55.6 mm travel length just in case the pinion gear does not collide with the end of rack structure.

This robot uses a power spring as an energy source due to the above advantages, and the power spring used for the proposed robot has an angular displacement of about 1800 degrees and a torque output of 0.05 Nm in the central flatter section. In addition, the power spring stores enough energy for 3.5 hops, excluding sections other than the central, flatter section. Furthermore, the winding motor continuously operates during hopping until the power spring is fully charged, so the number of hops is not limited.

### 2.3. Active Clutch

The active clutch mechanism is responsible for changing the driving direction of the rack to create a leap motion and adjusting the timing so that the leap is appropriate. The pinion gear connected to the power spring can only be driven in one direction due to the characteristics of the power spring. To solve this problem, the position of the actuation part, including the pinion gear, can be changed through the active clutch as shown in Figure 5. In addition, in order to change the drive direction of the rack using a single pinion, two racks must be placed in opposite positions. To implement this, a rectangular rack structure is designed with two racks facing each other. The rectangular rack is designed considering the pitch diameter and overall diameter of the pinion gear, so that the pinion gear cannot engage with both racks during clutching.

Figure 5a,b shows the rack being driven in the downward direction. As the active clutch rotates clockwise, the actuation part moves to the left, so the pinion gear engages the rack on the left. This causes the rack to move downward, allowing the robot to leap. Figure 5c,d shows when the rack is driven in the upward direction. When the active clutch motor rotates counterclockwise, the actuation part moves to the right. Therefore, the pinion gear engages the rack on the right side, resulting in the rack moving upward and retracting the hopping leg.

**Figure 6:**
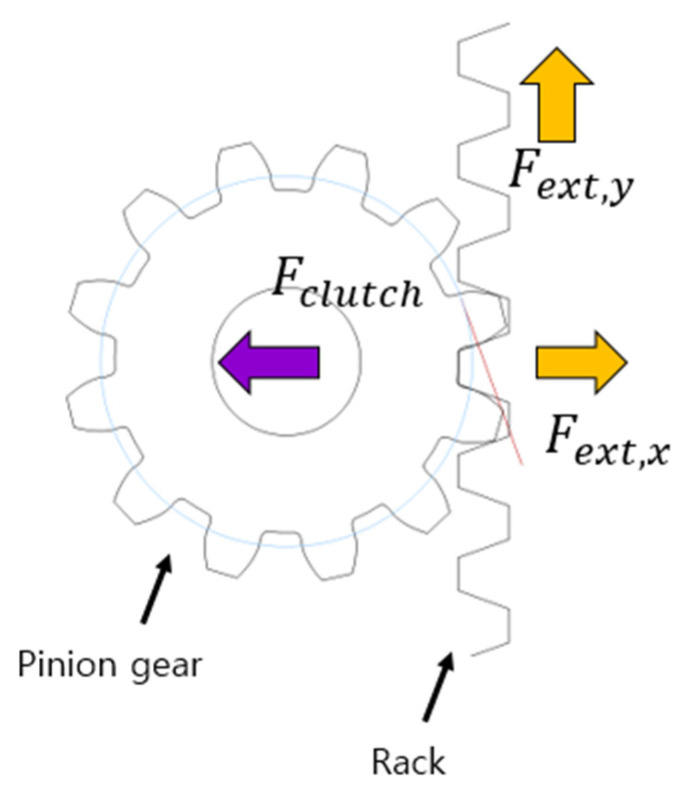
Force relation between the pinion gear and rack.

The basic purpose of the active clutch mechanism is to move the pinion gear to provide bi-directional drive. Therefore, the active clutch must operate smoothly. For example, the pinion gear must disengage well from the rack when the clutch motor is commanded. Failure to do so would result in a long clutching time, making it difficult to perform a well-timed leap. For smooth operation, the force from the clutch motor must be well transmitted to the pinion gear, which is related to the angle at which the pinion gear approaches the rack. Figure 6 shows the forces that affect the clutch. *F_c_* is the force exerted by the clutch motor and *F_ext_* is the force with which the rack holds the pinion gear. The clutch force must be equal to or greater than *F_ext_*_, *x*_, the force with which the rack holds the pinion gear, in order for the pinion gear to disengage from the rack smoothly. Therefore, it is necessary to minimise the *x*-direction component of *F_ext_*, and for this purpose, *F_ext_* is designed to be perpendicular to the clutch force so that *F_ext,x_* does not occur.

### 2.4. Hopping Control

**Figure 7:**
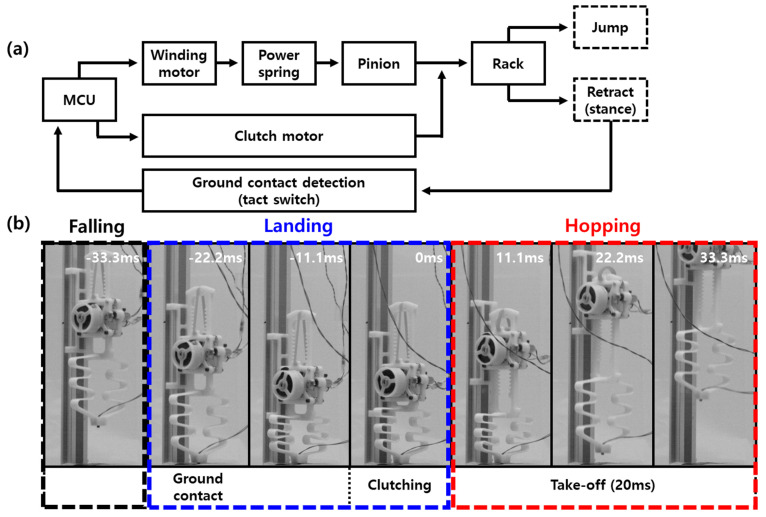
(**a**) Working algorithm of the active clutch and (**b**) the snapshots from landing to hopping.

In order to perform a successful hopping locomotion, it is important to command the clutch action at the right time. Figure 7a shows a schematic for controlling clutch timing. The clutch is based on a ground contact sensor attached to the tip of the foot. A tack switch is used to detect ground contact. When contact occurs, a high signal is generated and sent to the micro controller unit (MCU). Once the contact signal is received, it immediately commands the clutch motor to switch to energy release mode.

Figure 7b shows snapshots taken at 1000 frame per second (fps) from landing to leap. The first ground contact occurs at −22.2 ms, at which point a high signal from the tact switch is sent to the MCU. Based on this signal, the MCU gives a motion command to the clutch motor and the power spring switches the robot to energy-releasing mode. Due to the rotation speed of the clutch motor, clutching actually occurs at 0 ms, which means there is a delay of about 20 ms between the command from the MCU and the actual operation. To solve this problem, a passive spring connected in series is used in the foot. The passive spring in the foot serves to delay the time required for landing. Therefore, even though the actual clutching is delayed, the robot performs the leap at the right time. In Figure 7b, the toes lift off the ground at 11.1 ms and the robot takes off. After leaving the ground, the clutch motor gives a retraction command, causing the legs to retract. This process is repeated to achieve hopping locomotion.

## 3. Modeling

The proposed robot’s leap finishes its motion in a few hundred milliseconds. Therefore, dynamic modelling is essential to accurately analyze its behavior. Dynamic modelling provides a clear picture of the behavior of the robot from landing to leaving the ground. It quantitatively analyzes various characteristics such as the ground reaction force, the energy usage of the power spring, the degree of compression of the passive spring, and the velocity and acceleration of the robot. In addition, it investigates how changing the constant of the passive spring affects the leap.

### 3.1. Power Spring Analysis

**Figure 8:**
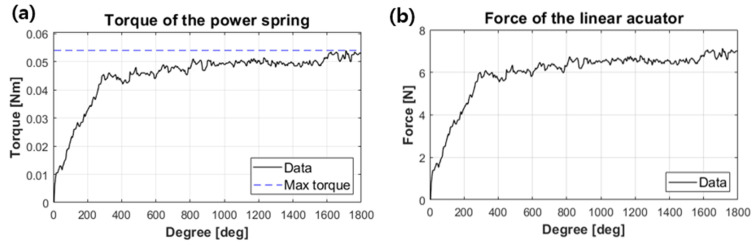
(**a**) The measured torque of the power spring and (**b**) the measured force of the rack.

Power springs have a central flatter section where the torque is almost constant with displacement. Therefore, compared to a typical torsion spring, energy storage is possible as long as the torque of the actuator required to store energy is higher than the torque of the central flatter section. The torque of a power spring is determined by the inner diameter of the barrel, the outer diameter of the central shaft that stores energy, and the spring thickness, width, and length. Figure 8a,b shows the torque of the power spring and the force curve measured on the rack connected to the power spring, respectively. It can be seen that the torque and force are relatively constant with the displacement after 300 deg., and the maximum torque and maximum force are 0.054 Nm and 7.13 N, respectively. The parameter of the power spring is as shown in Table 3.

### 3.2. Hopping Dynamics

**Figure 9:**
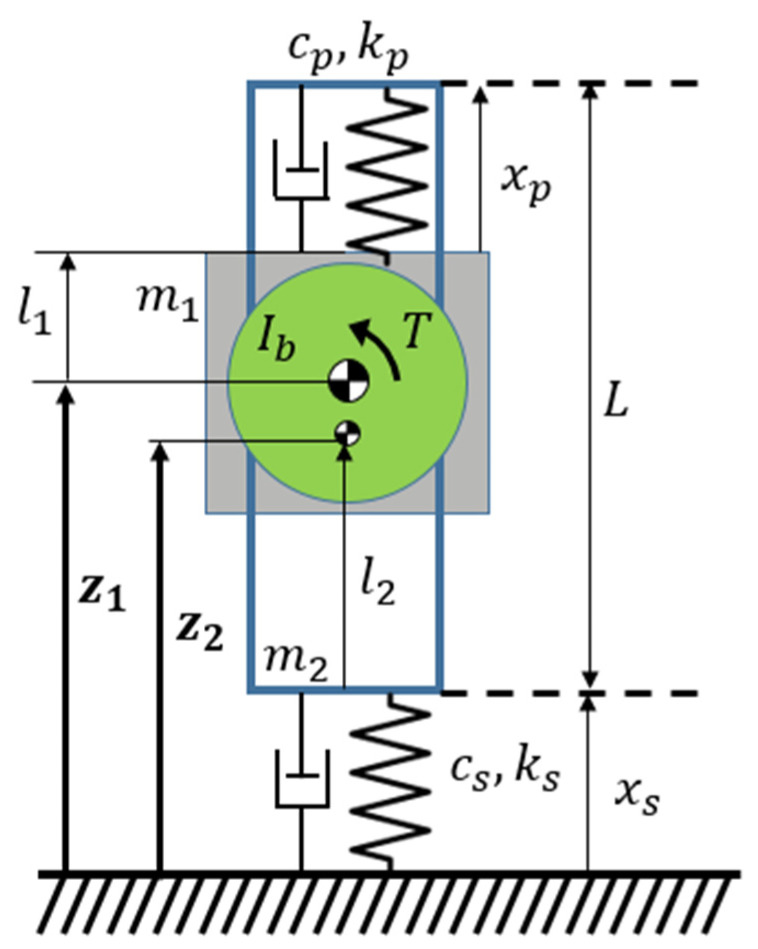
Schematic of the hopper’s model. Subscripts such as *p* and *s* denote the parallel spring and series spring, respectively.

The proposed hopping robot can be simplified to a model with two masses and two springs, as shown in Figure 9. From the model, we can derive the process of the robot from the time it touches the ground until it takes off. Let *m*_1_ and *m*_2_ be the masses of the rack and the actuation part, respectively. *z*_1_ and *z*_2_ are the distance from the ground to the center of gravity of each mass. *x_t_*, *x_c_*, *k_t_*, *k_c_*, *c_t_,* and *c_c_* are the displacement, stiffness, and damping coefficients of the tension and compression springs, respectively. *L*_1_ and *l*_2_ are the distance from the center of gravity to the springs, respectively, and *L* is the length of the rack. The parameter values for the analysis are as shown in Table 4. *X_t_* and *x_c_* are given as follows:

(1) $$x_{t}=z_{2}-z_{1}+L-l_{1}-l_{2}$$

(2) $$x_{c}=z_{2}-l_{2}$$

The forces from the tensile spring and compression spring, indicated as *F_t_* and *F_c_*, respectively, are given as follows:

(3) $$F_{t}=-k_{t}\left(z_{2}-z_{1}+C_{1}\right),{\;F}_{c}=-k_{c}\left(z_{2}-C_{2}\right),\;T_{p}\theta_{b}=\left\{\begin{array}{c} -F_{l}x_{t}\;:Before\;clutch\\ F_{l}x_{t}:\;After\;clutch \end{array}\right.$$

where *T_p_* is the torque from the power spring, $\theta_{b}$ is the angular displacement of the barrel, *C*_1_ = *L* − *l*_1_ − *l*_2_ − *x_t,I_*, *C*_2_ = *l*_2_ + *x_c,i_*, *x_t,i_*, and *x_c,i_* are the initial displacement of the tensile and compression springs.

*F_l_* has two values because it refers to the force at the end of the rack and changes the orientation of the actuator module before and after the clutch. The resulting equations of motion were analyzed numerically using the Matlab ODE45 function.

(4) $$\left[\begin{array}{cc} m_{1}+\frac{I_{b}}{r^{2}} & -\frac{I_{b}}{r^{2}}\\ -\frac{I_{b}}{r^{2}} & m_{2}+\frac{I_{b}}{r^{2}} \end{array}\right]\left[\begin{array}{c} \ddot{z_{1}}\\ \ddot{z_{2}} \end{array}\right]=\left[\begin{array}{cc} {-k}_{t} & k_{t}\\ k_{t} & {-k}_{t}{-k}_{c} \end{array}\right]\left[\begin{array}{c} z_{1}\\ z_{2} \end{array}\right]+\left[\begin{array}{cc} {-c}_{t} & c_{t}\\ c_{t} & {-c}_{t}{-c}_{c} \end{array}\right]\left[\begin{array}{c} {\dot{z}}_{1}\\ {\dot{z}}_{2} \end{array}\right]+\left[\begin{array}{c} k_{t}\left(L-l_{1}-l_{2}-x_{ti}\right)-m_{1}g\mp F_{l}\\ -k_{t}\left(L-l_{1}-l_{2}-x_{ti}\right)+k_{c}\left(l_{2}+x_{ci}\right)-m_{2}g\pm F_{l} \end{array}\right]$$

## 4. Results

### 4.1. Simulation Results

**Figure 10:**
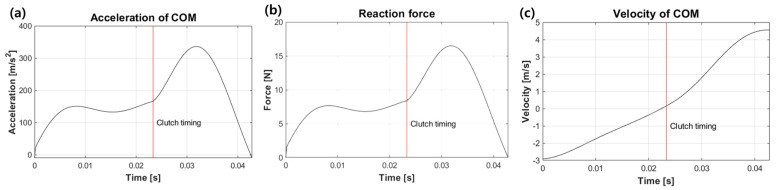
(**a**) Acceleration, (**b**) reaction force, and (**c**) velocity of the mass center, from the simulated results.

Figure 10 shows the simulation results based on the dynamics model of the hopping robot from the moment it contacts the ground until it leaps. Figure 10a–c refers to the acceleration, reaction force from the ground, and velocity of the robot’s center of gravity as a function of time, respectively. The robot falls with pre-stored energy in the power spring and stores additional energy after landing. At this point, it reaches its maximum energy, storing energy until its velocity theoretically approaches zero. The active clutch mechanism then changes the direction of travel of the rack, increasing its acceleration. The acceleration increases to approximately 320 m/s^2^ after the clutch timing and then gradually decreases until the reaction force is zero and the robot takes off. A single hopping process takes 22.8 ms from ground contact to clutch and 20 ms from clutch timing to leap, which results in 42.8 ms in total.

### 4.2. Effect of Passive Springs

**Figure 11:**
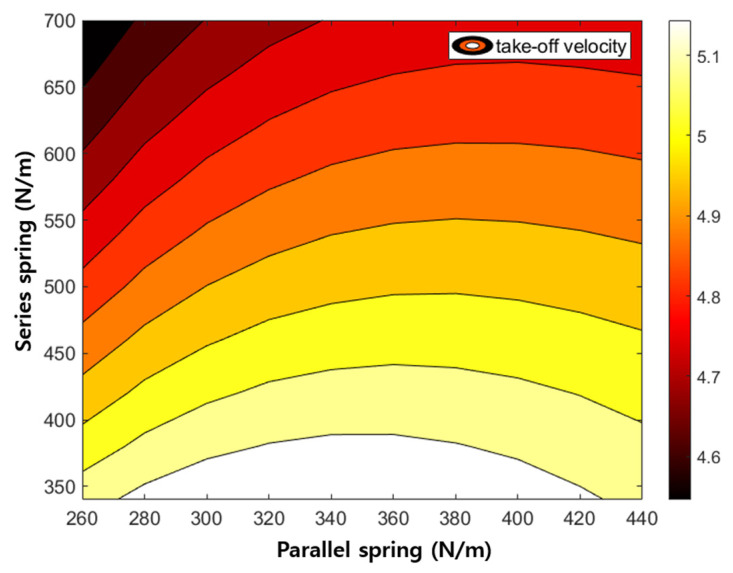
Take-off velocity by varying the spring constants of the parallel and series springs.

The proposed hopping robot has two passive springs. One tension spring is located above the actuation part and the other compression spring is located below. The spring constants of the two springs change the amount of energy recovered upon landing, which in turn affects the take-off speed. To understand the effect of the springs, the take-off velocity of the robot is investigated through the dynamic model by adjusting the spring constants of the passive springs as shown in Figure 11.

First, for the parallel spring installed at the top, the take-off velocity increases as the spring constant increases. For the series spring installed at the bottom, the take-off velocity decreases as the spring constant increases. This is because there is little compression displacement when the spring constant is large. While there is a clear limit to the ground reaction force applied by a hopping robot’s weight, if the spring constant is too large, it is difficult to store elastic energy because the spring is hardly compressed. When the spring stiffness is low, it is useful to store the elastic energy and accordingly, the hopper tends to take off with high speed as shown in Figure 11. However, if the stiffness is too low the spring over-compresses and the spring structure collides with each other. Considering this, the spring constants of the upper and lower springs are selected as 360 N/m and 540 N/m, respectively.

### 4.3. Hopping Performance

**Figure 12:**
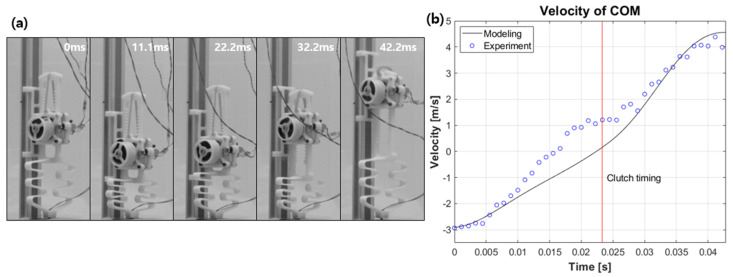
(**a**) High-speed images and (**b**) the corresponding velocity during landing.

To verify the actual hopping ability, a hopping experiment is conducted. Since the current robot has difficulty in controlling its balance, the hopping experiment is conducted using a linear guide. Figure 12a shows high speed images of the robot falling to the ground and jumping again. The time is expressed as 0 s from the time of landing on the ground. The hopping robot recognizes the ground contact through the tact switch attached to its toe when it lands on the ground. When the ground contact is recognized, the controller sends the energy release command to the clutch. At this time, a delay of about 20 ms occurs, and it can be seen in Figure 12a that the clutch command is actually executed at 22.2 ms. At 22.2 ms, the actual energy release occurs, and at 42.2 ms, the toe comes off the ground and performs a leap.

Figure 12b shows the velocity of the center of mass from video analysis based on modelling and experimental data. The negative velocity sign before the clutch timing is due to the center of mass moving downward as the passive spring located at the foot is compressed upon landing. As the center of mass continues to move downward and energy is released by the clutch, the center of mass moves upward and performs a leap. In Figure 12b, there are some differences between the modelled and experimental data, but after the point of clutching, they show a similar trend overall. The take-off speed at the time of leap results in 4.4 m/s for the experiment and 4.5 m/s for the simulation data.

**Figure 13:**
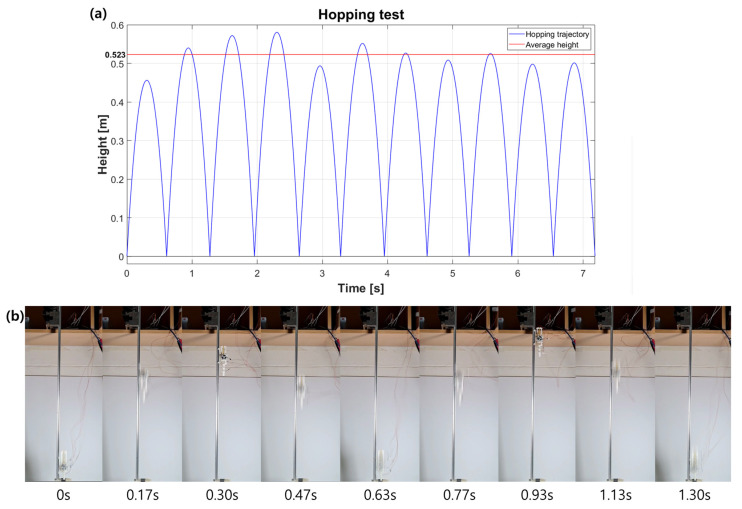
(**a**) Trajectory of the mass center during eleven hops. (**b**) Snapshots of the first and the second hopping.

Figure 13a shows the trajectory of the mass center plotted as a function of height over time for eleven consecutive hops. Figure 13b shows snapshots of the first two hops. Over the eleven hops, we observe an average height of 0.52 m and the maximum height of 0.58 m. In Figure 13a, we can see that the hopping heights are distributed within a certain range, from a low of 0.45 m to a high of 0.58 m. There are two main reasons for the difference in hopping height. First, the friction generated by the linear guide can affect the hopping height. The second reason is the delay in the clutch timing control. We confirmed that a delay of about 20 ms occurs, but the delay is not always constant. Therefore, the amount of energy stored in the passive spring at the time of hopping varies and may affect the hopping performance.

## 5. Conclusions

In this study, a small hopping robot is developed using a power spring-based linear actuator. The power spring stores energy even with a small amount of torque and stores energy efficiently because the spring has a central, flatter section. The power spring also has a displacement of more than 1800°, so it can store a lot of energy at once. The linear actuator is designed as a rack-and-pinion structure, and in order to achieve bi-directional linear motion, a rectangular rack and an active clutch mechanism are introduced. The active clutch mechanism solves the problem of limited directionality of the power spring and actively changes the driving direction of the rack. This ensures that the hopping robot can release energy at the right time during the short contact time with the ground. In order to use energy efficiently, the design is equipped with additional parallel and series springs. The parallel spring at the top serves to prevent energy loss and provide additional force when hopping, while the series spring at the tip allows the impact of landing to be converted into elastic energy and reused. In a result, the robot is 143 mm long, weighs 45.9 g, and has an average hopping height of 52.3 cm.

## Figures and Tables

**Table 2:** Mass budget of the proposed hopping robot.

Components	Quantity (EA)	Mass (g)	Mass Ratio (%)
Body frame	1	10.35	21.69
Winding motor	1	10.14	21.24
Power spring	1	12.34	25.85
Barrel	1	2.00	4.19
Pinion	1	0.48	1.01
Rack	1	4.68	9.81
Active clutch (motor, rack, pinion)	1	1.98	4.16
Series spring	1	3.57	7.48
Parallel spring	1	0.34	0.71
Guide	1	1.85	3.88
**Total**	**10**	**47.73**	**100.0**

**Table 3:** Detailed value for the design parameters.

Parameter	Value
Inner diameter of barrel	22.0 $\times \;10^{-3}$ m
Overall diameter of shaft	8.0 $\times \;10^{-3}$ m
Width of power spring	9.4 $\times \;10^{-3}$ m
Thickness of power spring	0.1 $\times \;10^{-3}$ m
Length of power spring	1.50 m

**Table 4:** Detailed value for the dynamic modeling.

Parameter	Value
$m_{1}$	0.0394 kg
$m_{2}$	0.0083 kg
$I_{b}$	0.172 $\times \;10^{-6}$ $kg\;m^{2}$
$k_{t}$	360 N/m
$k_{c}$	540 N/m
$c_{t}$	0.684 N s/m
$c_{c}$	0.529 N s/m
$l_{1}$	0.016 m
$l_{2}$	0.019 m
L	0.084 m
$x_{ti}$	0.0045 m
$x_{ci}$	0.053 m

## Data Availability

Data are contained within the article as figures and tables.
